# Does Allostatic Load in 50–89‐Year‐Olds Predict the Development of Frailty? Evidence From a National Longitudinal Study Over 12 Years

**DOI:** 10.1002/smi.3517

**Published:** 2024-12-15

**Authors:** Marco Arkesteijn, Rachel Bennett, Jennifer L. Davies, Rachel C. Sumner

**Affiliations:** ^1^ Department of Life Sciences Aberystwyth University Aberystwyth UK; ^2^ Education and Applied Sciences University of Gloucestershire Cheltenham UK; ^3^ School of Healthcare Sciences Cardiff University Cardiff UK; ^4^ Global Academy of Health and Human Performance Cardiff Metropolitan University Cardiff UK

**Keywords:** ageing populations, allostatic overload, healthy ageing, registered report

## Abstract

Frailty is characterised by a loss of function across several domains but is not an inevitable aspect of ageing and can be reversed with intervention. Determining those who are more likely to become frail before physical deficits become manifest will allow earlier intervention. One promising indicator of the potential for frailty is allostatic load, a physiological status associated with prolonged stress that is, characterised by multisystem dysfunction. Previous research has sought to understand the links between allostatic load and frailty, but has not yet explored whether allostatic load may be a predictive factor at younger ages and—if so—at what age it may be predictive. The present study sets out establish whether allostatic load can be used as a predictive indicator of frailty. Using the English Longitudinal Survey on Ageing (ELSA) data with an anticipated sample of 1500 people between 50 and 89 years old, time series analysis will determine if, and at what age, allostatic load may be predictive of pre‐frailty and frailty. The findings of these analyses may be supportive of early identification of frailty by establishing an age at which a diagnostic test for allostatic load may prove a critical indicator for future frailty.

## 1 Introduction

Ageing is associated with a progressive decline in physiological functions and metabolic processes (López‐Otín et al. 2023). This can result in frailty, an age‐related condition that is, characterised by a decline in functioning across multiple physiological systems. Frailty is associated with higher health care costs, increased dependence, and lower quality of life (Kojima et al. 2016). Frailty can be prevented and, once present, reversed (Dent, Morley, et al. 2019; Negm et al. 2019; Travers et al. 2019), but prevention appears to be more (cost) effective (Apóstolo et al. 2018; Dent, Martin, et al. 2019; Looman, Huijsman, and Fabbricotti 2019). Early identification of those on the trajectory towards frailty would therefore be highly valuable (Walsh et al. 2023).

Allostatic load is characterised by multisystem dysfunction resulting from the physiological wear and tear incurred from chronic stress. Although both frailty and allostatic load reflect dysfunction in physiological systems, allostatic load is resultant from prolonged stress and is therefore identifiable at any point during the life course (Crimmins et al. 2003), whereas frailty is generally identifiable in later life only. Prolonged allostatic load has been associated with declines in cognitive and physical functioning over time (Seeman et al. 2001), as well as age‐related degenerative conditions such as dementia (Twait et al. 2023) and Alzheimer's disease (Matos and De Souza‐Talarico 2019), providing a link between allostatic load and adverse outcomes in ageing. Several shared pathophysiological processes between allostatic load and frailty exist, including dysregulation to endocrine and immune systems, along with impaired or dysfunctional signalling in brain areas that also process psychological stress and initiate the physiological stress response (Clegg et al. 2013), suggesting a potential link between these two phenomena. As allostatic load can be present at any point during the life span, and is associated with dysregulations and dysfunctions of several systems also implicated in frailty, this might suggest that allostatic load could contribute to the development frailty, and therefore be useful as a prognostic indicator (Kuchel 2009). Moreover, as allostatic load is reversible through a variety of interventions aimed at reducing overall psychological and physiological stress (Guidi et al. 2020), it is ameliorable and therefore may also be an intervention point to delay onset of frailty.

Allostatic load reflects the accumulation of stressors across the life course (Crimmins et al. 2003) and frailty reflects the resilience to stressors and its impact on functioning in older age (Dent, Morley, et al. 2019). Despite the evidence for the multi‐system impact of allostatic load, and some of its relations with later age outcomes, remarkably little research has been conducted to understand if, how, and when allostatic load may predict the onset of frailty. There is evidence from cross‐sectional studies that allostatic load may be associated with earlier development of frailty (Fried et al. 2009; Guidi et al. 2020; Szanton et al. 2009). A similar relation has been suggested based on longitudinal research (Ding, Kuha, and Murphy 2017; Karlamangla et al. 2002), however, the design of these studies limits the conclusions that can be made. Ding et al. did not exclude participants with frailty at baseline, and only included participants over the age of 65 years. Co‐existence of frailty and allostatic load was likely, particularly in older age categories, and it cannot be determined if allostatic load preceded frailty. To establish whether allostatic load precedes the development of frailty in later life, a baseline with participants without frailty is warranted. Previous research (Shi et al. 2023) has also used overlapping concepts of allostatic load and frailty, by employing the frailty index, rather than frailty phenotype. Use of the frailty index means that similar, or even the same, variables will be used to quantify frailty and allostatic load as both rely on establishing the ratio of presence or absence of (medically diagnosed) of a self‐selected range of conditions (Cesari et al. 2014). In contrast, the frailty phenotype emphasises 5 domains of physical functioning (weight loss, weakness, exhaustion, slowness and low physical activity) specifically and exclusively (Cesari et al. 2014; Fried et al. 2001). Combined, this indicates it is currently unknown whether allostatic load is relevant as a prognostic indicator of frailty. Establishing such a relation would enable early identification of individuals who would benefit the most from interventions to prevent frailty, rather than these individuals only being identified when the decline in functioning becomes noticeable.

In summary, an association has been suggested between allostatic load and frailty both cross‐sectionally (Fried et al. 2009; Guidi et al. 2020; Szanton et al. 2009) and longitudinally (Ding, Kuha, and Murphy 2017; Karlamangla et al. 2002), however it is still not known whether allostatic load precedes the onset of frailty, and if it can predict frailty onset. Therefore, the aim of this research is to determine, among non‐frail individuals, if allostatic load is a predictor of future frailty. Using the existing data set of the English Longitudinal Study of Ageing (ELSA), allostatic load will be determined at baseline and frailty will be evaluated every 4 years thereafter for 12 years. It is hypothesised that allostatic load at baseline will be associated with a greater risk of developing frailty, and this relation will be present in adults who are young‐old (50–64 years) at baseline as well as those who are older (65–89 years) at baseline.

## 2 Methodology

### 2.1 Database Selection

Candidate longitudinal population survey datasets were identified using Gateway to Global Ageing Data (Gateway to Global Ageing Data), Google Scholar, and an initial scoping search of published literature on allostatic load or frailty. For consideration for inclusion in this study, a dataset had to meet the following criteria: At least 10 years of follow‐up; contain biomarker data; contain data sufficient to establish (pre)frailty; include participants aged 50–100 years; data stored in English. Thirty‐four datasets were identified and scrutinised to determine whether they incorporated variables that would enable frailty and allostatic load to be calculated, with the former preferentially based on functioning and the latter based on physiological biomarkers. Three datasets incorporated data that could be used to measure both the frailty phenotype model and allostatic load based on physiological biomarkers: The English Longitudinal Study of Ageing (ELSA), The Irish Longitudinal Study on Ageing (TILDA) and the Health and Retirement Study (HRS). At the time of writing (November 2023) access to the TILDA dataset was suspended, with no projected date of availability, so this database was excluded from consideration. HRS and ELSA were both deemed appropriate to address the research question, containing data from United States of America and England, respectively. ELSA was selected for the study, as the authors are based in the United Kingdom and have better understanding of socio‐demographic impacts and relevant co‐variates in the UK context than in the USA context.

### 2.2 Overview of Study

This longitudinal study will utilise secondary survey data collected over 12 years (2004–2016). Allostatic load will be determined for a population of non‐frail individuals using physiological biomarkers obtained in 2004/2005. Frailty status (non‐frail: 0 out of 5 indicators, pre‐frail: 1 or 2 indicators, frail: 3 or more indicators) will be determined using data from every available timepoint thereafter. The progression from non‐frail to pre‐frail and from non‐frail to frail will be evaluated using a discrete time event history analysis model. This will model the trajectory of developing (pre‐)frailty and determine the potential use of allostatic load as an early indicator.

### 2.3 Study Population

Participants in ELSA comprise a representative sample of people living in England aged 50–90+ years (Banks et al. 2021). Data have been collected in 10 waves spanning from 2002 to 2022. The earliest time point at which biomarkers were collected was Wave 2 (2004/2005). This will form the baseline timepoint of the current analysis. Variables required to evaluate frailty were also collected in Wave 2 and then subsequently in Wave 4 (2008/2009), Wave 6 (2012/2013), Wave 8 (2016/2017), and Wave 10 (2020/21). In these Waves, participants were followed up by computer‐assisted personal interviews and self‐completed questionnaires as well as a health examination nurse visit for collecting medical information. Individuals will be excluded if they are aged 90 years and older at Wave 2 because their age is top coded as ‘90’, if they have any frailty phenotype indicator at Wave 2, or if there are missing data for any of the variables required to determine allostatic load. All respondents gave informed consent as part of ELSA and consented to their data being used for secondary analyses. Ethical approval for ELSA was granted by the Multicenter Research and Ethics Committee.

### 2.4 Sample Size Estimation

It is not possible to know the available sample size until the data are accessed. The anticipated sample size available as baseline is larger than 2541 non‐frail participants, as this is the number of participants included in an existing analysis of wave 4, and it is likely that some participants were lost to follow up between wave 2 (the baseline for this study) and wave 4 (Leme and de Oliveira 2023). Attrition rate is however unknown over this period of time, although Leme and de Oliveira (2023) study indicated it was 39% between Waves 4 and 6.

The primary statistical analyses is a discrete time event history analysis model (also often called discrete time survival model). The study will be guided by the common rule of thumb is that there should be 10 events for every independent variable included in the model (Peduzzi et al. 1996). In this analysis there will be 12 independent variables (allostatic load plus 11 covariates explained in a subsequent section). This requires at least 120 (12 independent variables multiplied by 10 (Peduzzi et al. 1996)) participants at baseline to go onto experience the outcome event. Should the final sample size at baseline be insufficient, the exclusion criteria will be amended. Firstly, those individuals originally excluded due to omitted required covariates at Wave 2 will be included using mean/mode impute. If needed, subsequently, to no longer include ‘low physical activity’, since this is the only variable that deviates from Fried's original phenotype model. Low physical activity will then also not feature in the subsequent waves and frailty becomes a 4‐factor model. A 3‐factor model is the final resort, as employed previously by Ding, Kuha, and Murphy (2017), which would be based on the three factors that include the largest number of participants at baseline.

### 2.5 Frailty Definition

The two main approaches used to identify frailty comprise the relative presence of ageing‐related health conditions accumulated deficits, or the frailty phenotype (Cesari et al. 2014). The frailty phenotype model reflects the robustness and resilience of an individual's functioning and is selected for this study. The frailty phenotype reflects five indicators of daily functioning, comprising low gait speed, weak grip strength, low physical activity level, exhaustion, and unintended weight loss (Fried et al. 2001). A person exhibiting three or more indicators is considered to have frailty, whereas one or two indicators are classified as having pre‐frailty. Compared with the original phenotype model (Fried et al. 2001), four variables in ELSA are identical (low gait speed, weak grip strength, exhaustion, and unintended weight loss) and one will have to be adapted (low physical activity level).

Exhaustion will be derived from responses to two individual questions within the Centre for Epidemiologic Studies Depression Scale (CES‐D: Radloff 1977). The CES‐D is a 20‐item self‐report measure where respondents rate how often over the past week they experienced each listed symptom on a Likert scale ranging from zero (rarely or none of the time) to three (most or almost all the time). Exhaustion is considered present if a positive answer (rating 2–3) was provided to either of these two questions: ‘Felt that everything I did was an effort in the last week’ or ‘Could not get going in the last week’. A rating of one indicates some or little of the time.

Gait speed was assessed only in participants aged 60 years and over by measuring the time taken to walk a distance of eight feet at usual pace (Veronese et al. 2017). Low gait speed is defined using the mean gait speed of two walks at normal pace with the sex and height cutoffs suggested by Fried et al. (2001), even if the recommended walk distance was 15 feet (and not eight feet). Participants under the age of 60 were assumed to not have a low gait speed if they did not exhibit any of the other frailty phenotype criteria and were thus included. Those participants who could not perform the gait test owing to medical reasons are categorised as having low gait speed.

Grip strength was measured using a handheld dynamometer. Participants were instructed to squeeze the dynamometer as hard as they could. Maximum grip strength was determined as the highest value recorded after three attempts with each hand. Weakness was defined using the sex and body mass index (BMI) cutoffs suggested by Fried et al. (2001). Those not able to do the handgrip strength for medical reasons were considered as having weakness.

Weight change is defined as the difference in body mass (kg) between the current body weight relative to the body weight 4 years earlier (previous wave included in this study). Either the loss of ≥10% of body weight since previous time point or current Body Mass Index (BMI) < 18.5 kg/m^2^ is defined as the cut off criteria (Gale, Cooper, and Aihie Sayer 2014).

Physical Activity level is derived from a physical activity interview. Participants were asked about the frequency with which they did moderate exercise (e.g., gardening, cleaning the car, walking at a moderate pace, dancing, floor or stretching exercise). This question had four response options (more than once a week, once a week, one to three times a month, and hardly ever or never). Low physical activity will be defined as a response of ‘hardly ever or never’. This item was different than that proposed by Fried et al. (2001) because this set of criteria considers a different scale for defining low physical activity.

### 2.6 Allostatic Load Definition

A measure for allostatic load will be created using biomarker data from multiple domains, using the quantification methods outlined by Seeman et al. (1997) and using the system approach outlined by Richards, Maharani, and Präg (2023) to account for the likely impact of age‐related chronic illness on the various markers being assessed, and the inequity of number of markers across each system that they represent. The domains and markers to be used from the ELSA dataset include: immune system (C‐reactive protein [CRP], fibrinogen); metabolic system (total‐to‐HDL cholesterol ratio, triglycerides, glycated haemoglobin [HbA1c]); cardiovascular system (systolic blood pressure [SBP] and diastolic blood pressure [DBP]), and body fat deposition (BMI). These nine markers will be dichotomised into risk level (high or low) according to established criteria quartiles associated with overall risk generally, or with relevance to sex‐specific risk (Sanders et al. 2017). The allostatic load index will then be created by summing all of the risk levels per marker, where ‘high’ will be attributed a value of 1, and ‘low’ a value of 0, with higher total index scores indicating higher allostatic load. Each system will be assigned an allostatic load risk score, where each ‘high’ or ‘low’ attribution will be given equal weight, correcting for physical health conditions and medications that impact each system. This will result in a scale of 0–4, where 0 indicates no presence of allostatic load risk, up to 4, indicating allostatic load risk present for each system.

### 2.7 Covariates

#### 2.7.1 Demographic

To account for differences associated with both physical longevity and differentials between allostatic load and frailty, participant gender and age will be included. As allostatic load is understood to stabilise for a time around retirement (Juster, McEwen, and Lupien 2010), and age of retirement has also been found to be associated with frailty (Van der Elst et al. 2021), retirement is included as a covariate for the analyses.

#### 2.7.2 Socio‐Economic Status

Various aspects associated with social status are known to contribute to overall stress across the life course. As allostatic load is an index of accumulated physiological dysregulation due to chronic stress, level of education and wealth are included as covariates that are associated with social status. Level of education is split into a binary category of either having some qualifications or no qualifications at all. Wealth is assessed by creating a binary categorisation of the wealth deciles available within the dataset, with the bottom two deciles being grouped into a ‘low’ category and the other eight forming the ‘medium to high’ category.

#### 2.7.3 Health

Smoking behaviour, body mass index, and consumption of alcohol will be included as covariates due to their capacity to impact each of the systems being assessed within the allostatic load index. Cognitive impairment will also be incorporated into the analysis as a covariate because its association with frailty and that cognition could be included within the frailty phenotype. Cognitive impairment will be assessed using the Mini‐Mental State Examination (Folstein, Robins, and Helzer 1983), with a score between 0–30 and where a higher score indicates better cognitive performance.

#### 2.7.4 Social Context

To account for the contribution of the participants' social context into their overall health, a variety of covariates capturing elements of individual's social proximity and interaction will be used. Social integration is included as a means to assess levels of potential loneliness, a factor known to be strongly associated with both allostatic load and frailty (Juster, McEwen, and Lupien 2010; Kojima et al. 2016). Social integration is calculated using the method outlined by Ding, Kuha, and Murphy (2017), using a combined score of five items: partnership status (living with spouse/partner or not); three measures of frequency of contact (with children, with family, with friends) by either physically meeting, speaking with on the telephone, or writing emails or letters; and whether or not they are members of a social club, or other similar type of organisation or society. Social support is determined similarly according to Ding et al.'s conceptualisation, combining both a lack of positive support from friends and family and the presence of negative support also. Lack of positive support is measured using any responses that indicate disagreement to questions in the self‐complete questionnaire module about partnerships and friendships, including ‘How much do they really understand the way you feel about things?’, ‘How much can you rely on them if you have a serious problem?’, and ‘How much can you open up to them if you need to talk about your worries?’. The presence of positive support is measured using responses that indicate agreement or high frequency to statements such as ‘How much do they let you down when you are counting on them?’ and ‘How much do they criticise you?’.

### 2.8 Statistical Analysis

Analyses will be run separately to evaluate each of the two outcome variables: frailty and pre‐frailty. The sample for the analysis based on the pre‐frail outcome variable will exclude frail individuals. Based on life table estimates, the survivor functions for each outcome by age group and gender will be plotted as a function of time since baseline (often referred to as survivor curves). This will give an overview of the proportion of the sample at baseline who remain non‐frail/non‐prefrail over time and differences in these proportions by age group and gender.

Discrete‐time event history models will then be used to model the odds of experiencing frailty/pre‐frailty, whilst accounting for time since baseline. A discrete‐time approach will be used because frailty/pre‐frailty is only measured once per survey wave—so the exact timing of become frail/pre‐frail is not known. Binary logistic regression modelling will be performed on the data in person‐period format, where each observation represents a participant's data for each wave ‘at risk’ of experiencing the outcome.

In the first models, allostatic load and time will be included as covariates to examine whether there is significant association between allostatic load at baseline and propensity to experience frailty/pre‐frailty after accounting for time. In the subsequent models, demographic characteristics, socio‐economic characteristics, health characteristics and social support characteristics will be added in turn to examine whether controlling for these factors changes the relationship between allostatic load and propensity to experience frailty/pre‐frailty. In the final models, time, allostatic load, demographic characteristics, social characteristics, health characteristics and social support characteristics will all be included.

This modelling approach will first be applied to the whole sample to explore the association between allostatic load and propensity to experience frailty/pre‐frailty amongst all 50–89 year olds. In the second phase, the sample will be stratified by age group and the modelling repeated for each age group separately (with the same covariates)—that is, firstly 50–59 year olds only, secondly 60–69 year olds only, thirdly 70–79 year olds only and lastly 80–89 year olds only. This will reveal whether the predictive power of allostatic load for future frailty/pre‐frailty varies by age. As a robustness check, the modelling will also be repeated for men and women separately (with the same covariates) to examine whether the predictive power of allostatic load for future frailty/pre‐frailty varies by gender. If differences are found by age and/or gender, the original (unstratified) model will be re‐run with an interaction term between age and/or gender and allostatic load.

#### 2.8.1 Missing Data

To account for missing data in the covariates, cases where less than 5% of respondents have missing data will be excluded, or if more than 5% have missing data, a ‘missing data’ category will be created. An analysis of the characteristics of participants with and without missing data will be conducted to describe any impacts of removing these cases on the representativeness of the sample. The number of cases lost to follow up and the reasons for this will be studied and reported. The data will then be reshaped into person‐period form where each row represents an observation succeeding baseline and before the individual leaves the risk set—either by experiencing the outcome variable being study (frailty or pre‐frailty) or because they were lost to follow up.

## Conflicts of Interest

The authors declare no conflicts of interest.

## Data Availability

The data that support the findings of this study are available in English Longitudinal Study of Ageing at https://www.elsa‐project.ac.uk/data‐and‐documentation. These data were derived from the following resources available in the public domain: UK Data service, https://beta.ukdataservice.ac.uk/datacatalogue/series/series?id=200011.
